# Immunomodulatory Function of Soluble Dietary Fiber: Toward Application for Prevention and Treatment of Food Allergies

**DOI:** 10.1007/s11882-026-01292-3

**Published:** 2026-09-29

**Authors:** Masako Toda, Andrea Wangorsch, Yui Yajima, Yingnan Guo, Chaoqi He, Stefan Vieths, Stephan Scheurer

**Affiliations:** 1 Laboratory of Food and Biomolecular Science, Graduate School of Agricultural Science, Tohoku University, Sendai, Japan; 2 Molecular Allergology, Paul-Ehrlich-Institut, Langen, Germany; 3 Present Address: Current Address: Cancer Center, Department of Breast Surgery, Zhejiang Provincial People’s Hospital (Affiliated People’s Hospital), Hangzhou Medical College, Hangzhou, China

**Keywords:** Dietary fiber, Food allergy, Pattern recognition receptors, Gut microbiome

## Abstract

**Purpose of Review:**

Gastrointestinal dysbiosis is implicated in the disruption of immune homeostasis and the pathogenesis of food allergies. Consequently, dietary fibers have gained attention as key molecules modulating microbial balance and immunity. This review aims to explore the immunomodulatory functions of dietary fibers commonly found in the human diet, including pectin, inulin, β-glucan, and mannan.

**Recent Findings:**

Dietary fibers affect the immune system through two primary pathways: direct interaction with pattern recognition receptors and indirect action via microbial metabolism. In the indirect pathway, the gut microbiome utilizes these fibers as an energy source to produce a diverse range of metabolites. Notably, these fiber-derived processes exhibit a dual nature in food allergies, showing potential to either suppress or, in some cases, promote allergic inflammation.

**Summary:**

Understanding the unique physicochemical and physiological characteristics of individual types of fibers is important for the rational development of dietary-based prevention and treatment strategies in food allergies.

## Introduction

Food allergy (FA) represents a global public health challenge. There is a strong demand for innovative prevention and treatment strategies based on the fundamental mechanisms underlying pathogenesis. Evidence has suggested that dysbiosis, the qualitative and quantitative alterations in the gut microbiome, is involved in the disruption of immune homeostasis and the pathogenesis of allergies [1–3]. This microbial and metabolic dysregulation may impair gut barrier function and Treg induction and facilitate allergic inflammation by triggering innate signals and subsequent adaptive Th2-type immunity [1–3].

Considering the central role of dysbiosis in FA pathogenesis, dietary fiber has emerged as a dietary component for microbial modulation [4, 5]. For practical purposes, dietary fiber is broadly classified into insoluble fiber and soluble fiber. Insoluble fiber does not dissolve in water, increases stool bulk, and promotes intestinal motility by stimulating the tissues. Soluble fiber dissolves or is transformed into a gel-like substance in water and gastrointestinal fluids. Therefore, soluble dietary fiber is generally more easily fermented by gut microbiome as an energy source, leading to the production of various metabolites. Evidence has accumulated that certain types of organic acids including short-chain fatty acids (SCFAs) and other microbial metabolites such as bile acids and tryptophan metabolites from the gut microbiome contribute to immune modulation [6–8]. In addition, some dietary fibers bind to pattern recognition receptors (PRRs) and thereby potentially modulate the response of immune cells [9, 10]. The binding to PRRs can be a key factor in the action of dietary fiber, alongside the metabolites produced by gut microbiome.

This review addresses the potential of soluble dietary fiber for the prevention and treatment of FA. While various types of soluble fiber exist, we focus on inulin, pectin, β-glucan, and mannan. These four major dietary fibers are present in diverse food sources and are also widely utilized in the food industry due to their versatile functional properties. Their potential to bind PRRs and effects on the gut microbiome, subsequent metabolite induction, and immune modulation in FA are discussed. The complex interplay of key metabolites, including SCFAs, bile acids, tryptophan metabolites, and lactic acid, is also addressed briefly.

## Mechanism of Immune Modulation Via Microbial Metabolites

### Short-Chain Fatty Acids (SCFAs)

**Fig. 1:**
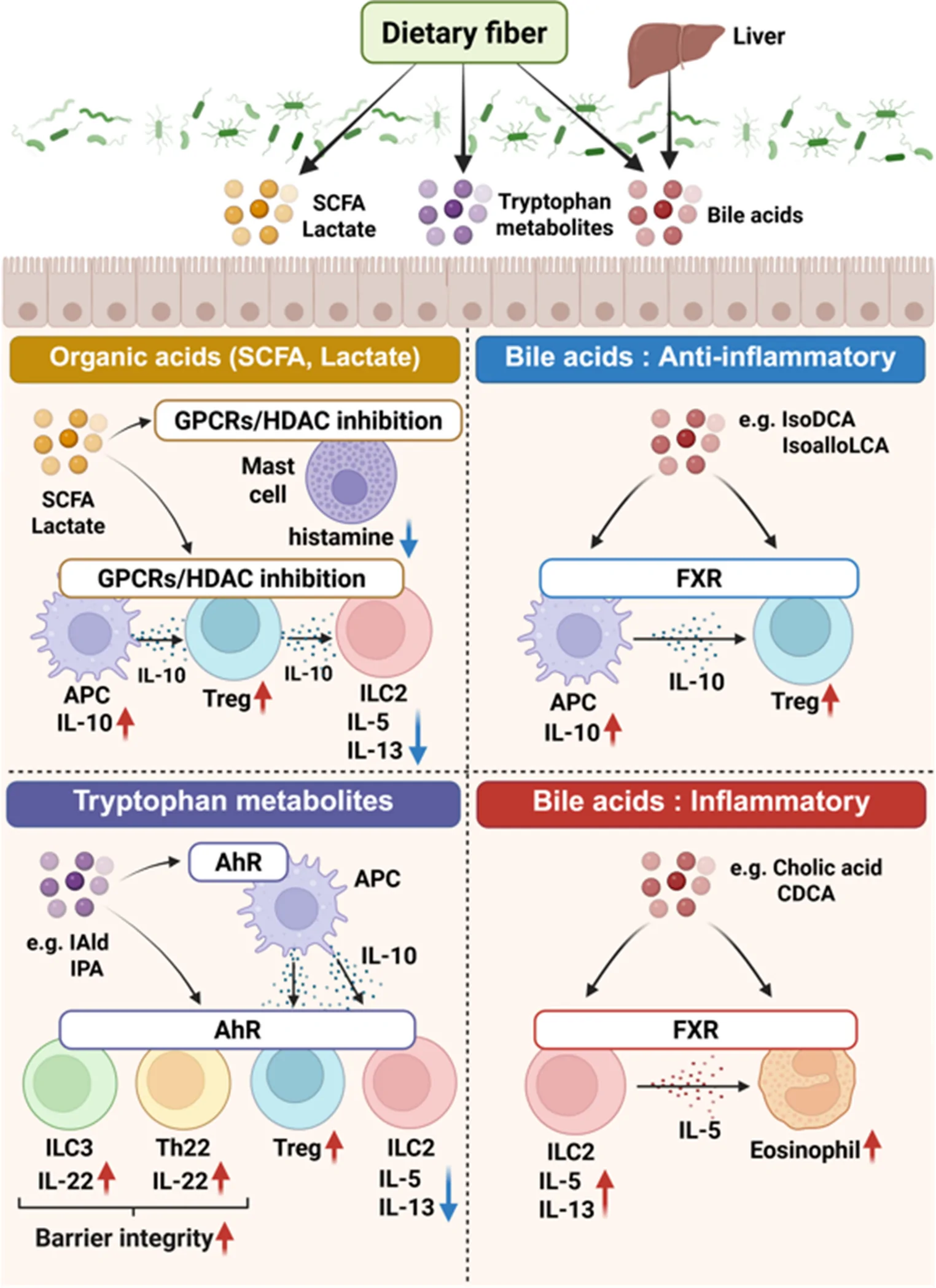
Schematic overview of immune modulation via microbial metabolites derived from dietary fibers. Evidence has accumulated that certain types of dietary fibers induce immunomodulatory metabolites via the gut microbiome. Immune cells express specific receptors for these metabolites. SCFAs bind to GPR41, GPR43, and/or GPR109a. Lactic acid binds to GPR81. Tryptophan metabolites act as ligands for the AhR. These interactions induce immune cells with regulatory phenotypes such as tolerogenic APCs and Tregs, and inhibit other effector cells such as mast cells and ILC2s involved in allergic responses and inflammation. SCFAs also act as HDAC inhibitors and thereby facilitate the differentiation of Tregs. Bile acids exhibit a dual role in immune modulation; bile acids such as cholic acid promote type 2 inflammation via the induction of ILC2s and eosinophils, whereas several bile acids such as isoDCA or isoalloLCA induce Tregs via FXR signaling in DCs or metabolic remodeling (adapted from ref [5])

SCFAs exert their effects on various cell types through two primary modalities: acting as ligands for G-protein-coupled receptors (GPCRs) including GPR41, GPR43 and GPR109a and functioning as potent histone deacetylase (HDAC) inhibitors (Fig. 1) [6, 11–13]. SCFA-mediated GPR109a signaling or HDAC inhibition promotes the induction of tolerogenic dendritic cells (DCs) and macrophages (Mφ) [14–16]. Butyrate and propionate suppress mast cell activation via HDAC inhibition or GPR109a pathways [17, 18]. Butyrate attenuates the activation of group 2 innate lymphoid cells (ILC2s) by inhibiting the master transcription factor GATA3 and the secretion of type 2 cytokines via HDAC inhibition [19, 20]. GPR43-driven phosphorylation cascades and butyrate-induced epigenetic remodeling of the *Foxp3* locus via HDAC inhibition facilitate the differentiation of Tregs [16, 21, 22]. SCFAs also act on intestinal epithelial cells via GPR41 and GPR43 to reinforce the gut barrier by up-regulating mucin and tight junction proteins, which may prevent food allergen entry [6, 12]. Taken together, SCFAs have the capacity to maintain immune tolerance and suppress type 2 allergic responses.

### Bile Acids

Primary bile acids, i.e., cholic acid and chenodeoxycholic acid (CDCA), are synthesized from cholesterol in the liver and secreted into the duodenum for the absorption of dietary lipids. Dietary fiber affects the enterohepatic circulation of bile acids by inhibiting their reabsorption in the ileum. This influences microbial conversion into secondary bile acids and alters the bile acid composition in the colon. Bile acids bind to several membrane receptors, e.g., Takeda G-protein-coupled receptor 5 (TGR5) and leukemia inhibitory factor receptor (LIFR), and nuclear receptors, e.g., farnesoid X receptor (FXR) and vitamin D receptor (VDR) [8, 23, 24]. Importantly, bile acids appear to exert context-dependent effects on immune responses, capable of either suppressing or promoting allergic inflammation depending on their specific molecular profiles. For instance, isodeoxycholic acid (isoDCA), a secondary bile acid derived from the gut microbiota, increases Foxp3 expression in T cells by acting on DCs via the FXR [25]. Isoallolithocholic acid (isoalloLCA), a secondary bile acid, increases Treg differentiation by inducing mitochondrial reactive oxygen species [26]. In contrast, increased levels of bile acids including cholic acid and CDCA induce Th2 cytokine production by ILC2s and drive eosinophilia via FXR in intestinal stromal cells [27]. Several studies have indicated that the bile acid profile in allergic patients differs from that of healthy individuals [7, 28–30].

### Tryptophan Metabolites

Dietary fiber intake significantly influences the microbial breakdown of tryptophan into various indole derivatives. These metabolites include indole-3-aldehyde (IAld), indole-3-propionic acid (IPA), and indole-3-lactic acid (ILA), which act as strong ligands for the aryl hydrocarbon receptor (AhR) [31, 32]. AhR signaling in DCs induces a tolerogenic phenotype and promotes the expansion of Tregs and IL-22-producing type 3 innate lymphoid cells (ILC3s) [33, 34]. IL-22 is pivotal for maintaining epithelial homeostasis and upregulating tight junction proteins and antimicrobial peptides, as well as promoting mucus production, which together protect against allergen translocation in the intestine [35, 36]. In addition, Li et al. showed that AhR inhibits Th2 cytokine production by ILC2s, thereby suppressing type 2 inflammation in *C. rodentium*-infected mice [37]. By facilitating the production of indole-based AhR ligands, dietary fiber may strengthen the intestinal barrier and contribute to a state of tolerance.

### Lactate and Microbial Cross-Feeding

Lactate is produced by various gut bacteria, such as *Bifidobacterium* and certain *Bacteroides* species, and is involved in immune modulation. Colonic DCs and Mφ express GPR81, a lactate receptor on the cell surface that maintains immune tolerance and regulates colonic inflammation [38, 39]. Lactate also crosses cell membranes via specific transporters, such as monocarboxylate transporters (MCTs), which allow it to exert its intracellular effects. Several studies in cancer immunology have shown that lactate induces regulatory factors in APCs and fosters a Treg-enriched environment [39–41]. In the gut, lactate also serves as a substrate for secondary fermenters, e.g., *Faecalibacterium prausnitzii*, which convert it into butyrate through microbial cross-feeding [42, 43]. This synergistic interaction supplies butyrate, potentially reinforcing the induction of tolerogenic APCs and Foxp3^+^ Tregs, thereby contributing to protection against FA.

## Anti-Allergic Potentials of Dietary Fibers

### Pectin

#### Structure and Origin

**Fig. 2:**
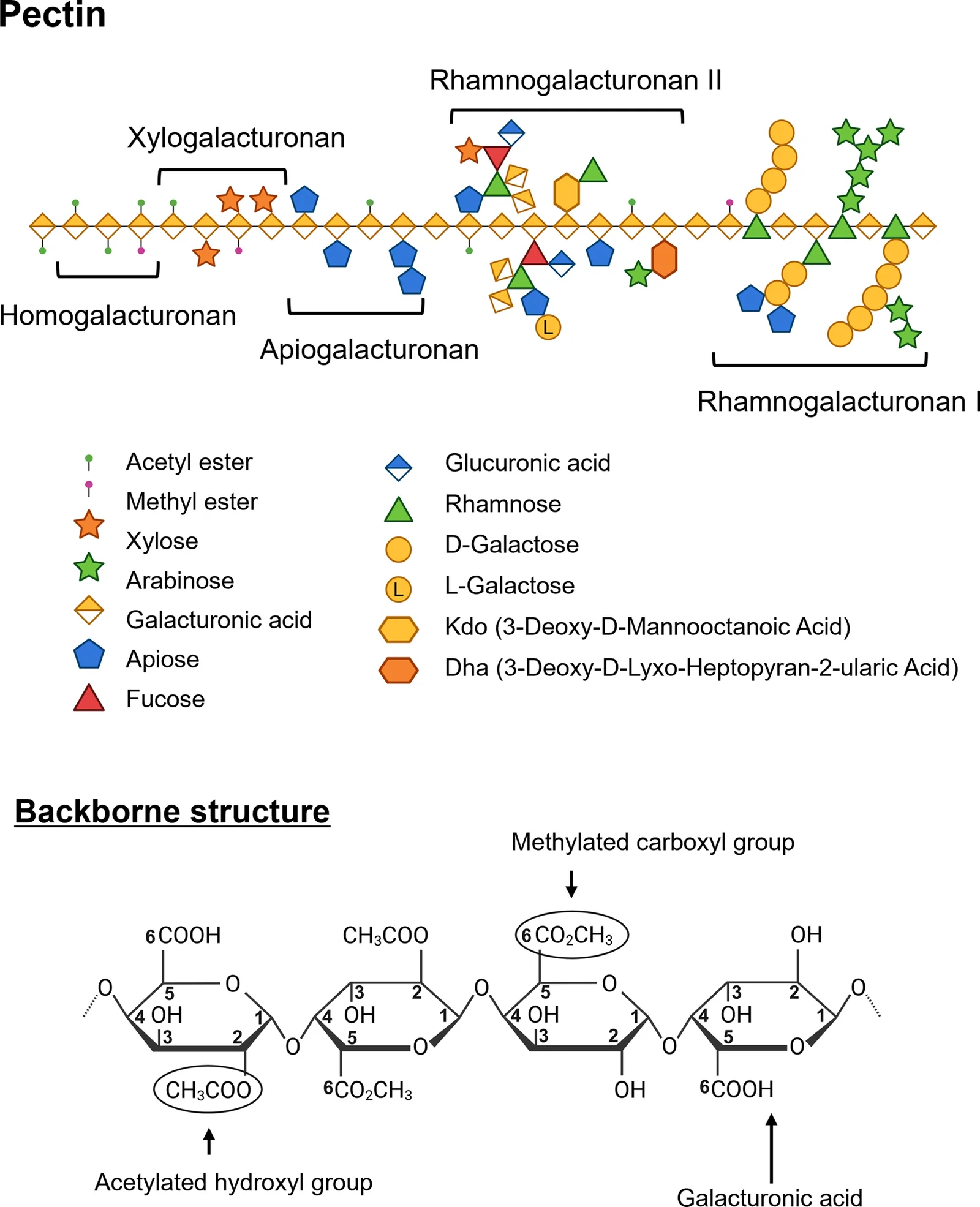
Primary structure of pectin. Aschematic representation of the major pectin domains (HG, RG-I, RG-II, XGA, and AGA) and their backbones. The diagram highlights that the galacturonic acid units are methyl-esterified at the C-6 position and acetylated at the C-2 or C-3 position (adapted from ref. [44, 45])

Pectin refers to a group of soluble fibers found in the cell walls and intercellular tissues of certain plants such as apples and citrus fruits. The main backbone of pectin is a linear chain of α-(1,4)-D-galacturonic acid units. Pectin consists of five domains: rhamnogalacturonan I (RG-I), rhamnogalacturonan II (RG-II), xylogalacturonan (XGA), apiogalacturonan (AGA), and homogalacturonan (HG) (Fig. 2) [44–46]. The composition and structure of branches in these regions, particularly in rhamnogalacturonans, vary depending on the source of pectin.

Furthermore, pectin can be structurally divided into two groups: high-methoxy pectin (HMP) and low-methoxy pectin (LMP), based on the degree of methyl esterification (DE) of D-galacturonic acid [46]. Most natural pectin is HMP, whereas LMP is more common in processed pectin. In addition to methyl esterification, the hydroxyl groups at the C-2 or C-3 positions of the galacturonic acid units can be acetylated (Fig. 2). While citrus and apple pectin typically have a very low degree of acetylation, pectin from sources such as sugar beets or potatoes is often highly acetylated [47].

#### Interaction with PRRs

Although the immunomodulatory effects of pectin in most reports are attributed to the gut microbiota and the fermentation process, pectin also interacts directly with intestinal and mucosal cells, promoting immune cell responses via PRRs [48]. Pectin exerts different immunomodulatory effects that are caused by the distinct structural features and binding characteristics of the polysaccharide [49, 50]. Citrus LMP binds and blocks the TLR1/2 pathway [49, 51]. Citrus HMP binds to LPS, modifying LPS binding to TLR4 in Mφ and potentially competing with LPS-mediated TLR4 activation [52, 53]. Pectin RG-I is considered to enhance immune responses [54–57]. RG-I containing arabinogalactan side chains has been suggested to modulate APC activation by affecting the galectin-3 signaling pathway [58–60].

#### Anti-Allergic Effects in Experimental Models

While our previous review article covered the studies on the anti-allergic effects of pectin until 2021 [61], the present review highlights new findings. In particular, several studies published over the past few years have analyzed the correlation between pectin structure and its anti-allergic effects.

Our group showed that dietary supplementation with apple HMP, but not with citrus LMP, reduces mast cells and eosinophils in the gut of naive mice [62] and suppresses the development of peach-derived lipid transfer protein (LTP)-induced FA in a murine model [63]. Specifically, apple HMP reduced several primary bile acids, including cholic acid, and increased secondary bile acids such as 7-ketolithocholic acid and ursodeoxycholic acid (UDCA). UDCA exerts pleiotropic effects, including anti-inflammatory and cytoprotective activities [64]. These findings suggest that pectin exerts its anti-allergic effects at least in part through the modulation of intestinal bile acids.

Besides the degree of methyl esterification (DE/DM), the degree of blockiness (DB) representing the clustering of non-esterified galacturonic acid units, appears to influence pectin’s immunomodulatory potential. Beukema et al. demonstrated that long-term, low-dose supplementation with specific citrus pectin induces Rorγt^+^ Tregs in mice; this effect was observed with both high-DB/low-DM and low-DB/intermediate-DM pectins, but was not observed with the low-DB/low-DM pectin. This effect was attributed to an enriched gut microbiota, e.g., *Lachnospiraceae NK4A136*, which enhances the production of AhR-activating tryptophan metabolites [65].

Polygalacturonic acid (PGA), which lacks both methyl esterification and acetylation and possesses a low degree of polymerization, also exhibited anti-allergic effects in a murine model [66]. In addition, the highly branched RG regions of pectin could play a crucial role in immune modulation [67, 68]. Due to the presence of acetylation and other complex structural variations, establishing a definitive correlation between pectin architecture and its physiological effects remains highly challenging.

#### Human Intervention Studies

The impact of pectin on the human gut microbiota and its metabolic activities has been well-summarized in recent literature [69, 70]. Several key commensal bacteria, including *F. prausnitzii*, *Prevotella copri*, and various Bifidobacterium and Bacteroides species, are known to actively degrade pectin. While their metabolic utilization and expansion often depend on the structural characteristics of the pectin source, these taxa also exhibit common functional responses to the galacturonic acid backbone shared across different pectins [69, 70].

While clinical intervention trials evaluating pectin specifically in allergic patients remain limited, a recent study by Gómez et al. provided valuable translational insights [71]. The effects of a prebiotic intervention with apple HMP and citrus LMP were investigated using a multi-omics approach in patients with LTP-mediated peach allergy and a placebo-treated control group [71]. Patients received 20 g of commercial pectin daily for 2 months in a randomized DBPC intervention study. Although no significant differences in LTP-specific skin prick test results and specific IgE levels were detected after intervention, more than 50% of patients in both pectin groups showed an increased tolerance to peach LTP by DBPCFC. Targeted proteomics revealed significantly different expression of 10 proteins after intervention in comparison with placebo. The two pectins modulated different sets of proteins, but a reduction of IL-13 and IL-24 was observed in both groups, whereas LMP specifically decreased IL-33 and IL-17C, and HMP suppressed TSLP and IL-4. The modulation of the microbiome and bile acids was specific to the type of pectin, e.g., LMP enriched *Roseburia* intestinalis, whereas HMP enriched *Bifidobacterium* and *F. prausnitzii*; however, both pectins similarly increased *Sutterella* and reduced branched SCFAs including isobutyric acid and isovaleric acid. Integrated multi-omics profiling revealed that LMP and HMP induce distinct biomarker networks. The authors concluded that pectin treatment may represent a promising therapeutic approach for LTP allergy, although confirmation in larger clinical studies is required [71].

In addition to standard dietary pectins, Modified Citrus Pectin (MCP) has also been investigated [72]. MCP is a special form of LMP derived from citrus peels, processed to reduce its molecular weight, thereby enhancing systemic absorption [72]. MCP has been extensively evaluated in clinical trials for its potential to inhibit galectin-3, a protein associated with cancer progression and fibrosis [73, 74]. Its clinical application in FA and other allergic conditions remains unexplored.

### Inulin

#### Structure and Origin

**Fig. 3:**
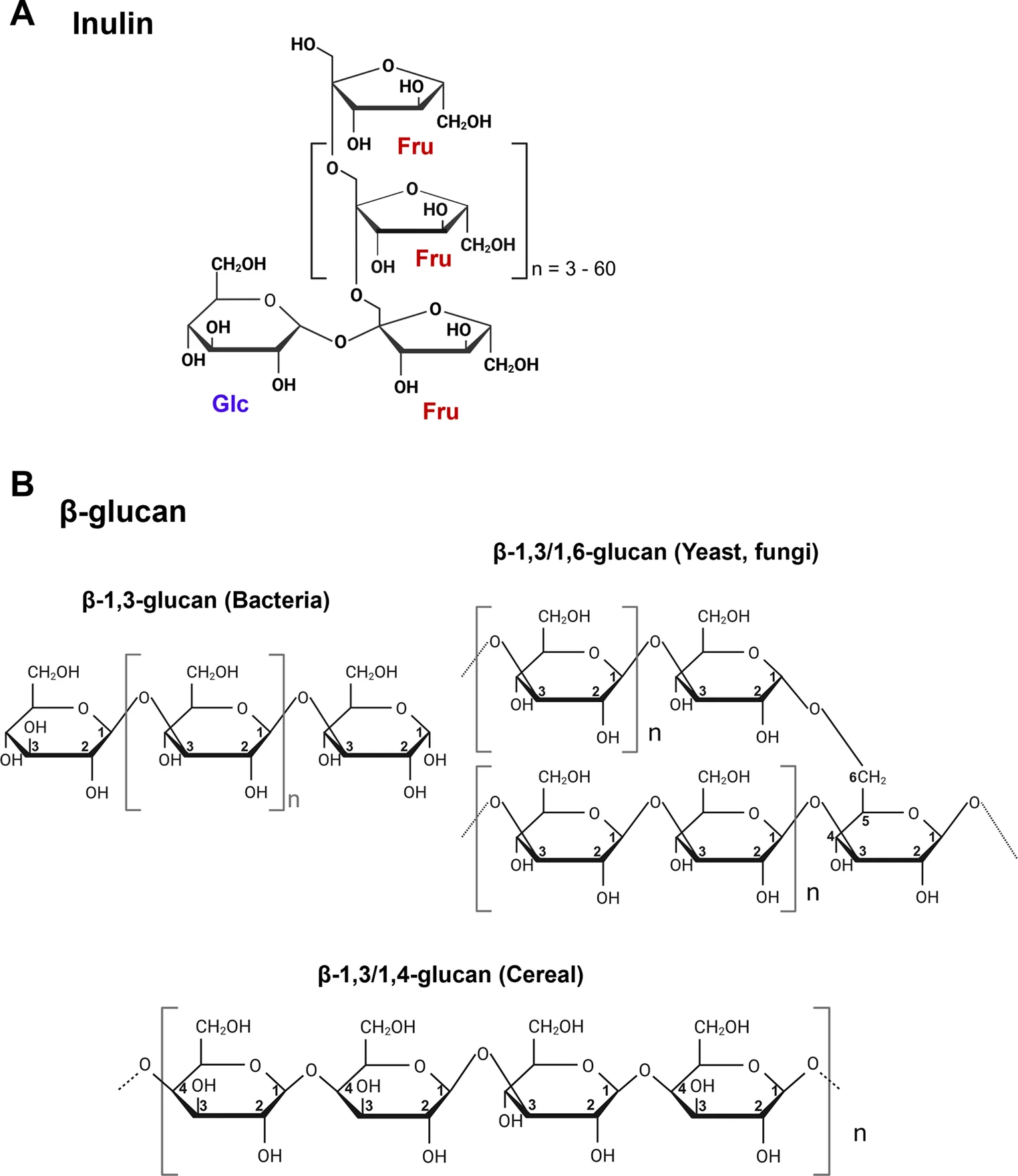
Primary structures of inulin and β-glucan. **A** Structure of inulin, consisting of a linear chain of fructose (Fru) units linked by β-(2→1) bonds, typically terminating in a glucose (Glc) unit. **B** Structures of β-glucans from different sources: linear β-1,3-glucan from bacteria; β-1,3/1,6-glucan from yeast and fungi, featuring a β-1,3-linked backbone with β-1,6 branches; and linear β-1,3/1,4-glucan from cereals, composed of β-1,4-linked segments separated by β-1,3 linkages. (adapted from ref. [116])

Inulin is one of the most commonly consumed soluble fibers globally. It is a linear fructan composed of β-(2→1)-linked fructose units (typically 3 to 60 units) with an α-(1→2)-linked terminal glucose (Fig. 3A) [75–78]. As a storage carbohydrate, inulin is abundant in various plants, particularly within the Asteraceae (Compositae) family, e.g., roots of chicory (*Cichorium intybus*) and tubers of Jerusalem artichoke (*Helianthus tuberosus*) [76, 77]. While natural chicory inulin is commonly used to enhance the dietary fiber content of processed foods, it contains a broad spectrum of fructooligosaccharides (FOS; also known as oligofructose) with a degree of polymerization (DP) of < 10, as well as long-chain inulin (DP ≧ 10). Inulin produced via enzymatic synthesis is also available, though it primarily consists of FOS [76, 77].

#### Interaction with PRRs

Early studies suggested that inulin and FOS might act as TLR2 ligands or mild TLR4 ligands. TLR4 deficiency reduced the effect of inulin and FOS on cytokine production in rodent monocytes, although only a high concentration of inulin and FOS (5 mg/mL) triggered cellular responses in that study [79]. A study using human PBMCs showed that short-chain fructans induced the production of the anti-inflammatory cytokine IL-10 via TLR2 ligation [80]. However, compared with other soluble fibers such as α-mannan or β-glucan, direct interactions between inulin and PRRs remain less defined, suggesting that inulin exerts its immunomodulatory functions primarily through indirect pathways driven by microbial metabolism rather than direct PRR engagement.

#### Anti-Allergic Effects in Experimental Models

Several studies have shown the beneficial effects of an inulin-rich diet or intragastric administration of inulin in murine models of FA [81–83]. The mechanism of action has been explained by the promotion of beneficial bacteria in the gut, which in turn alters the Th1/Th2 balance and/or induces Tregs in lymphoid tissues. Furthermore, maternal intake of galactooligosaccharides (GOS)/inulin decreased the development of FA in offspring in a mouse model [84]. Oral immunotherapy (OIT) using an inulin gel/allergen complex reduced allergic development in food-allergic mice, alongside elevated ileal levels of guanosine, a microbial metabolite potentially promoting Th1 and Treg subsets [85].

Conversely, high inulin intake may promote Th2 inflammation. Arifuzzaman et al. showed that feeding mice with a 30% inulin diet expands intestinal Bacteroides producing bile salt hydrolase (BSH) and elevates microbiome-derived bile acids including cholic acid. Bile acids then trigger stromal cells to produce IL-33, and induce ILC2s to release IL-5, leading to eosinophilia in the colon and lungs [27, 86]. Xie et al. showed that oral administration of a high dose of inulin exacerbates intestinal inflammation in food allergic mice. It was suggested to be caused by excessive SCFA accumulation, potentially leading to lower luminal pH and localized epithelial barrier dysfunction [81]. These findings suggest that the safety and efficacy of fermentable fibers depend on the host’s innate immune status, microbiome composition, and, critically, the amount of inulin intake.

#### Human Intervention Studies

The International Scientific Association for Probiotics and Prebiotics (ISAPP) defines a prebiotics as “a substrate that is selectively utilized by host microorganisms conferring a health benefit.” Within this category, inulin-type fructans are recognized for promoting beneficial bacteria such as *Bifidobacterium* and *F. prausnitzii* in healthy individuals [87, 88]. In human intervention studies with inulin, an increase in SCFAs, particularly acetate and butyrate, has also been observed [87, 88]. However, the influence of chain length and source on their efficacy remains debated.

Double-blind, placebo-controlled (DBPC) randomized clinical trials have explored the impact of inulin-type fructans on FA. Maternal GOS/inulin supplementation from 20 weeks of gestation until delivery did not prevent FA or atopic dermatitis in the offspring [89]. Another study evaluated an amino acid-based formula supplemented with synbiotics (FOS/inulin together with *B. breve M-16V*) in infants with IgE-mediated cow’s milk allergy (CMA) [90]. Although the synbiotics shifted the fecal metabolome and increased *Bifidobacterium* metabolites after six months, their effect on the acquisition of milk tolerance remains unconfirmed.

While clinical trials on inulin monotherapy targeting FA are lacking, a potential benefit has been observed in another allergic condition. In a three-way crossover trial for stable asthma, 12 g/day of inulin improved asthma control and reduced sputum eosinophils, whereas inulin plus probiotics or placebo did not [91].

### β-Glucan

#### Structure and Origin

β-glucans are non-starch polysaccharides, either soluble or insoluble depending on their structure, composed of D-glucose units with β-glycosidic linkages, and widely present in the cell walls of cereals, bacteria, and fungi, but also in other organisms such as algae and insects. β-Glucans include β-1,3-glucan, β-1,3/1,6-glucan, and β-1,3/1,4-glucan (Fig. 3B).

##### β-1,3-glucan

Bacteria produce β-1,3-glucan characterized by a linear structure. Curdlan is β-1,3-glucan derived from Agrobacterium. It features a massive linear structure composed of 3,000 to over 12,000 D-glucose residues interconnected through β-1,3-linkages [92, 93]. Due to its high purity and well-defined linear structure, curdlan is widely utilized in immunological research as a gold-standard stimulus for investigating β-glucan-mediated immune responses. Paramylon is a well-known β-1,3-glucan derived from the microalga *Euglena gracilis* [94, 95]. Its degree of polymerization varies depending on growth conditions, typically ranging from 700 to several tens of thousands of D-glucose units. β-1,3-glucan is also produced from yeast cell walls by treatment with a specific cleaving enzyme, e.g., zymolyase [96, 97].

##### β-1,3/1,6-glucan

β-1,3/1,6-glucan consists of a linear β-1,3-glucan backbone with β-1,6-linked side chains and is primarily found in fungi. The structure of these branches varies significantly; β-1,6 branches in fungal hyphae are typically short and widely spaced, whereas those in yeasts such as *Saccharomyces cerevisiae* are longer and more complex [98–100]. The presence of β-1,3/1,6-glucans in seaweed (e.g., laminarin) is also well-characterized [94].

##### β-1,3/1,4-glucan

Cereals such as barley, oats, and rye contain β-1,3/1,4-glucan (mixed-linkage glucans). These are composed of linear chains of β-1,4-linked glucose segments, typically cellotriosyl or cellotetraosyl units, interconnected by solitary β-1,3-linkages [101, 102].

#### Interaction with PRRs

The immunological function of β-1,3-glucan and β-1,3/1,6-glucan has been investigated intensively. These glucans bind to specific receptors, including Dectin-1 and complement receptor 3 (CR3), which are primarily expressed on the surface of APCs, as well as lactosylceramide (LacCer) expressed on neutrophils.

##### β-1,3-glucan

The major receptor of β-1,3-glucan is Dectin-1 [103]. This receptor recognizes the β-1,3-linked D-glucose backbone via its C-type lectin-like domain (CTLD), and plays an important role in host defense against fungi [103, 104]. While the minimum recognition unit of Dectin-1 is a β-1,3-glucotriose, the initiation of a biological immune response requires much longer β-1,3-glucan polymers, typically 10 or more residues [105]. Additionally, Dectin-1 exhibits higher affinity for the helical conformations of long-chain β-1,3-glucan polymers, particularly the triple helix [103].

##### β-1,3/1,6-glucan

Dectin-1 recognizes the β-1,3 backbone of β-1,3/1,6-glucan, whereas Dectin-2 recognizes β-1,3/1,6-glucan by detecting the structural arrangement of high-density branching [104, 106]. In addition, CR3 and LacCer recognize β-1,3/1,6-glucan polymers. CR3 consists of CD11b and CD18 [107, 108]. The lectin-like domain (LLD) of CD11b (CR3) specifically recognizes the structural arrangement of β-1,3/1,6-branching areas [107]. This interaction is spatially distinct from the complement-binding site, allowing CD11b to serve as a dual-specificity receptor that facilitates the phagocytosis of fungal particles in APCs.

##### β-1,3/1,4-glucan

The immune system appears to exhibit lower sensitivity to β-1,3/1,4-glucan. β-1,3/1,4-glucans are primarily found in cereals such as oats and barley. This low responsiveness may be an evolutionary adaptation to avoid chronic immune activation by ubiquitous dietary components [109].

#### Anti-Allergic Effects in Experimental Models

##### β-1,3-glucan and β-1,3/1,6-glucan

Certain types of β-glucans have been reported to exhibit experimental inhibitory effects on the development of FA. Water-soluble, low-molecular-weight β-1,3/1,6-glucan induced IFN-γ production in the spleen and intestine, and inhibited allergic reactions in allergen-sensitized mice [110, 111]. Intragastric administration of lentinan (from shiitake mushrooms) reduced anaphylactic symptoms [112]. Conversely, a diet supplemented with β-1,3-glucan, but not with β-1,3/1,6-glucan from *S. cerevisiae, * promoted the development of allergic features in OVA-induced FA mice [113]. This exacerbating effect of β-1,3-glucan was attributed to its adjuvant effect on enhancing type 2 immunity by activating APCs.

The key to resolving these contradictions may lie in the physicochemical properties of β-glucans. While insoluble particulate glucans effectively activate Dectin-1 signaling in APCs, smaller soluble glucans bind to Dectin-1 without activating the cells, acting instead as antagonists [114–116]. By blocking chronic activation of Dectin-1 induced by environmental fungal antigens, these soluble antagonists suppress the subsequent secretion of pro-inflammatory cytokines, shifting the local immune environment away from Th2/Th17 induction. Future therapeutic strategies may need to prioritize the use of soluble or specific molecular-weight glucans that favor the induction of immune tolerance, while avoiding high-molecular-weight insoluble forms that may trigger Th2/Th17-mediated inflammation [117].

##### β-1,3/1,4-glucan

Intragastric administration of oat β-1,3/1,4-glucan reduced allergic reactions in an OVA-induced FA model [118]. This protective effect was accompanied by an increased abundance of *Lachnospiraceae* and its associated genera, as well as elevated levels of acetate, butyrate, and total SCFAs in the colon. Antibiotic treatment abolished the beneficial effects of oat β-1,3/1,4-glucan, suggesting that its anti-allergic action is mediated through a prebiotic effect on the gut microbiota.

#### Human Intervention Studies

Clinical evidence regarding the effects of β-glucans in patients with FA remains elusive. A few intervention studies have been reported in allergic rhinitis and asthma. An earlier DBPC study assessed the effects of daily supplementation for 4 weeks with Wellmune WGP^®^ β-1,3/1,6-glucan (WGP) from yeast (250 mg/day) in ragweed-allergic patients, and reported a reduction in nasal and eye symptoms [119].

In a recent international, multicenter DBPC trial, treatment with Imunoglukan P4H^®^, β-1,3/1,6-glucan from *Pleurotus ostreatus* (pleuran), plus vitamin C in asthmatic children improved asthma control and reduced the frequency of asthma exacerbations after 24 weeks of treatment. Furthermore, it resulted in a higher proportion of patients achieving well-controlled asthma status after 48 weeks of treatment, compared with the vitamin C-only group [120]. Detailed analyses regarding the impact on immunological parameters and the gut microbiome remain limited in these studies.

### Mannan

#### Structure and Origin

Mannans are mainly composed of D-mannose. Mannans can be largely divided into α- and β-types, which consist of α- and β-linkages, respectively.

##### α-Mannan

**Fig. 4:**
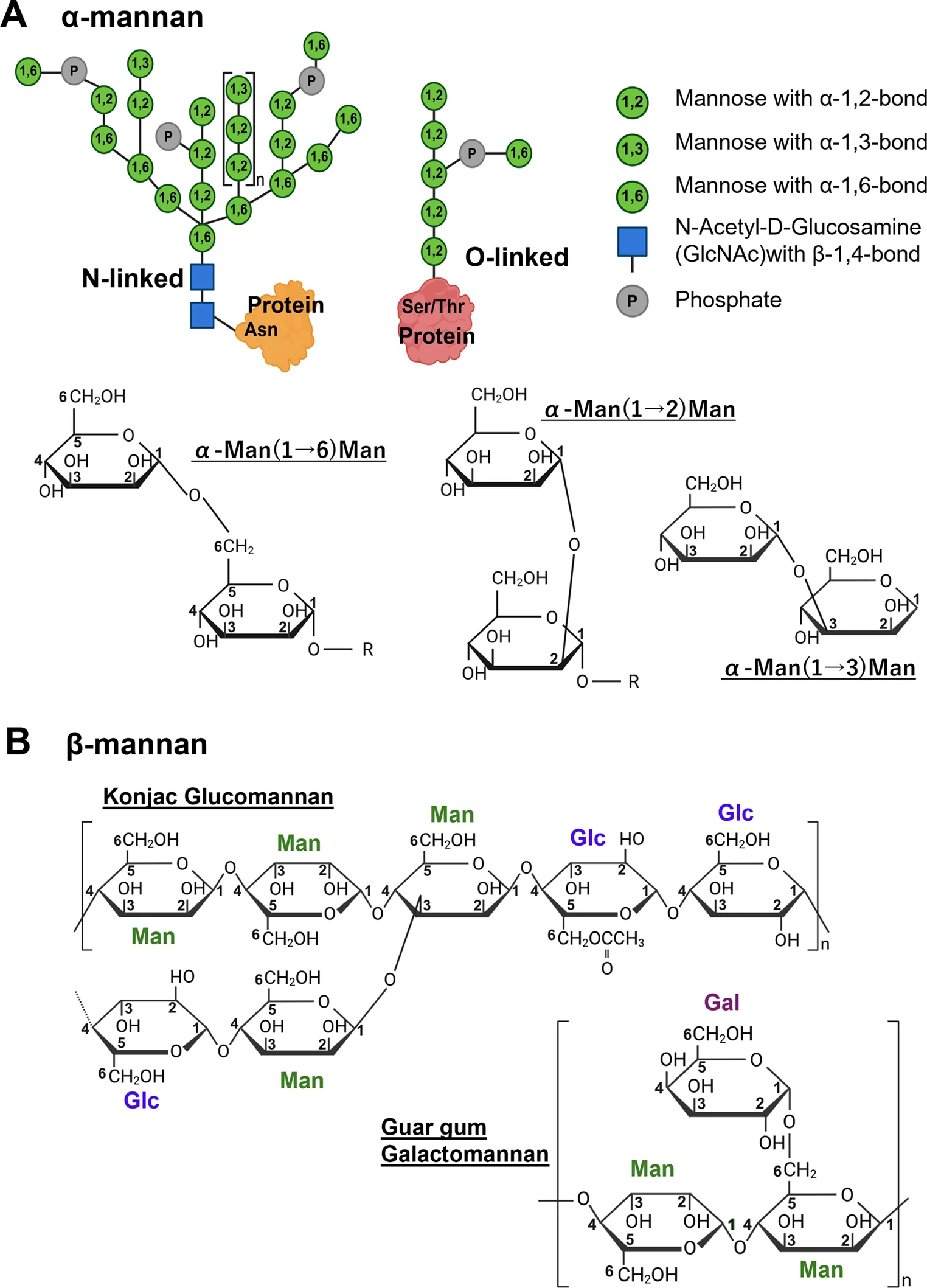
Primary structures of α-mannan and β-mannan. **A** Top: Schematic representation of N-linked and O-linked α-mannan structures attached to proteins via asparagine (Asn) and serine/threonine (Ser/Thr) residues, respectively (adapted from ref. [121, 122]). Green circles indicate mannose residues with specific glycosidic linkages (α-1,2-, α-1,3-, and α-1,6-bonds), and gray circles containing “P” represent phosphomannose groups. Bottom: Representative chemical structures of specific mannosidic linkages. **B** Detailed chemical structures of complex β-mannan derivatives (konjac glucomannan and guar gum galactomannan), showing the specific configurations of glucosyl (Glc), galactosyl (Gal), and mannosyl (Man) units with their corresponding glycosidic linkages (adapted from ref. [123–125])

α-Mannans are key structural components of fungal cell walls. While their specific structures vary across different fungal species, those derived from yeasts, particularly *S. cerevisiae*, are of interest for potential applications as functional dietary fibers and immunomodulators. In yeasts, α-mannans typically exist as complexes of mannoproteins with N-linked glycosylation at asparagine residues and O-linked glycosylation at serine/threonine residues (Fig. 4A) [121, 122]. While O-linked mannans consist of short and linear chains of 1 to 5 residues, the structure commonly known as “yeast α-mannan” refers to the N-linked type. This N-linked mannan is composed of an α-1,6-mannoside backbone with approximately 200 mannose units, which is heavily branched with α-1,2- and α-1,3-mannoside linkages (Fig. 4A).

##### β-Mannan

Mannans with β-linkages primarily serve as storage polysaccharides in plants. These include glucomannans and galactomannans. Structurally, these fibers consist of a backbone of β-1,4-linked D-mannosyl residues (Fig. 4B) [123–125]. The major botanical source of glucomannan is the konjac tuber (*Amorphophallus konjac*). Glucomannans feature a backbone of mannose and glucose, randomly substituted with O-acetyl groups. These acetyl groups, along with its high molecular weight, contribute to the high viscosity and gelling properties of konjac glucomannan, which significantly influence gut transit time and the rate of microbial fermentation.

Galactomannan contains a linear β-1,4-mannan backbone with α-1,6-linked galactose side chains (Fig. 4B). While Aspergillus and other fungi possess galactomannan in the outer layer of their cell walls, the majority of galactomannan consumed as dietary fiber is derived from botanical sources, such as legume seeds. The galactose-to-mannose ratio (G/M ratio) of galactomannans varies depending on their botanical sources, which influences solubility, viscosity, and gelling properties.

#### Interaction with PRRs

##### α-Mannan

Prominent receptors involved in the recognition of fungal mannans include Dectin-2, DC-SIGN, and the mannose receptor (MR; CD206). They are expressed on myeloid cells, such as DCs and Mφ.

Dectin-2 recognizes internal α-1,2-linked mannan in structures of fungal mannans via its carbohydrate recognition domain (CRD) [126, 127]. This receptor lacks an internal signaling motif and requires FcRγ, which contains an ITAM that triggers the Syk-CARD9 pathway and leads to the activation of APCs [128]. Involvement of Dectin-3 in the recognition of α-mannan has also been reported [129].

The MR recognizes terminal mannose units, primarily targeting the complex branched structures of α-mannans through multivalent binding across its CRD [130]. The MR is an endocytic receptor and internalizes ligands including pathogens in APCs for enhanced CD8^+^ T-cell activation [131].

DC-SIGN forms a tetramer on the cell surface and recognizes the terminal Man (α-1-2) Man residues at the distal ends of highly branched N-glycans [132]. Its signaling involves the Raf-1 pathway and modulates TLR-induced responses. DC-SIGN is also an endocytic receptor that delivers ligands to MHC class II loading pathways for efficient CD4^+^ T-cell activation [133].

##### β-Mannan

Recent studies have shown that TLR4 and the MR recognize certain types of β-mannans, e.g. konjac glucomannan and galactomannan from *Sesbania cannabina* or *Antrodia cinnamomea* [134–137]. These β-mannans exerted a stimulatory effect on murine Mφ and DCs via TLR4 [135–138]. However, Feng et al. have shown that chemically acetylated konjac glucomannan, but not its unmodified natural form of the molecule, is stimulatory to Mφ [139]. Regarding galactomannan derived from *Cyamopsis tetragonoloba* (guar gum), both stimulatory and inhibitory effects on APCs have been reported [140]. These apparent discrepancies in the immunological function of β-mannans could arise from differences in their botanical source, molecular weight, and isolation process.

#### Anti-Allergic Effects in Experimental Models

##### α-Mannan

α-Mannan from *S. cerevisiae* has garnered attention as an effective adjuvant in AIT. Allergoids coupled with α-mannan generated tolerogenic human DCs, which in turn induced functional Tregs through the programmed death-ligand 1 (PD-L1) pathway upon DC-SIGN engagement [141]. Nanoparticles displaying OVA and mannoprotein containing α-mannan effectively reduced anaphylaxis in a murine model [142]. Dietary supplementation with *S. cerevisiae-*derived α-mannan has been shown to suppress the development of allergic asthma in a murine model [143]. Information regarding the effects of dietary supplementation with α-mannan specifically in FA models remains limited.

##### β-Mannan

Early studies showed that dietary supplementation with pulverized konjac glucomannan suppressed IgE levels and allergic symptoms in murine models of allergic dermatitis [144, 145]. Le et al. showed recently that konjac glucomannan reduces *Salmonella*-induced colonic injury by modulating the TLR2-NF-κB signaling pathway, which was associated with restoring the abundance of *Muribaculaceae* and *Lactobacillus* [146].

Application of polysaccharides as allergen conjugates is expected to enhance the safety and efficacy of AIT by blocking IgE epitopes. OIT with a conjugate of peanut proteins and guar gum galactomannan exerted therapeutic effects in a murine model of peanut allergy [147]. It is also worth noting that the physiological impact of fiber could be double-edged. In a murine model of non-alcoholic steatohepatitis (NASH), a diet high in galactomannan (15%) exacerbated liver damage, whereas a citrus pectin diet at the same dosage was less hepatotoxic [148]. While these findings from animal models involving the gut–liver axis require cautious interpretation when extrapolating to humans, they suggest the existence of a potential therapeutic window and highlight the importance of carefully evaluating the safety of high-dose, fiber-specific interventions across different metabolic contexts.

#### Human Intervention Studies

##### α-Mannan

α-Mannan-conjugated allergoids for subcutaneous immunotherapy are in a phase of advanced clinical development [149–151]. Developed by Inmunotek, these therapies target primary allergens including birch pollen, grass pollen, and house dust mites, thereby addressing diverse patient populations. The prebiotic properties of mannan are not yet well characterized. *Bacteroides thetaiotaomicron* and *Bacteroides ovatus* have been identified as utilizing yeast mannan in humans [152, 153]. These bacterial species possess gene loci, known as Polysaccharide Utilization Loci (PULs) that express extracellular glycoside hydrolases such as α-mannosidases and endo-α-1,6-mannanases. The resulting cleavage of complex α-mannan into manno-oligosaccharides facilitates syntrophic cross-feeding with bacteria such as *Bifidobacterium* [152].

##### β-Mannan

Clinical evidence evaluating the effects of glucomannan and galactomannan in allergic patients remains limited. A Phase I/II study of OIT using a Cry j 1-galactomannan conjugate in patients with Japanese cedar pollinosis demonstrated improvements in both total symptom and medication scores during the cedar pollen season [154]. Immunologically, these effects were associated with increased allergen-specific IgG4 production and IL-10 secretion from PBMCs.

Plant-derived β-mannan is primarily degraded by *B. ovatus* and *Roseburia intestinalis* using PULs containing endomannanases [155, 156]. *F. prausnitzii*, a well-known butyrate producer, has been identified as a cross-feeder of the resulting β-1,4-glucomanno-oligosaccharides [157, 158]. While β-mannan potentially exerts prebiotic effects, its distinct physical properties may limit research efforts; while some galactomannans exhibit high solubility, others with low galactose substitution, and most particularly glucomannan, exhibit high viscosity. Hydrolyzed forms of β-mannan, such as mannooligosaccharides, have been reported to exert anti-inflammatory effects in patients with inflammatory bowel disease (IBD) [159]. Utilizing β-mannan derivatives could be a strategy to maximize their prebiotic effects while overcoming the solubility and viscosity constraints inherent in native fibers.

## Conclusions, Limitations, and Future Perspective

To transition these findings from experimental models to clinical practice, several key challenges need to be addressed.

1. Precision treatment: Future research may increasingly focus on precision dietary interventions in which fiber structures are selected according to an individual’s baseline gut microbiota, although the clinical utility of this approach remains to be established. Clinical outcomes are likely to depend on the distinct biological signatures and metabolic potential of the host’s resident microbes.

From a physicochemical perspective, the adverse effects observed in animal models following high-dose intake may reflect an imbalance between substrate availability and the metabolic capacity of the gut microbiota. When the amount exceeds the host’s microbial processing capacity, a rapid shift in metabolic flux, for instance, characterized by the excessive production of lactic acid and SCFAs, or an altered bile acid composition, could theoretically lower luminal pH or trigger pro-inflammatory signals that challenge epithelial and immune integrity.

While these mechanisms remain to be fully elucidated in human clinical settings, they highlight the importance of identifying safe and effective dosage regimens for dietary fiber intervention. Experimental studies have shown that high-dose inulin may exacerbate type 2 inflammation through the bile acid–IL-33–ILC2 axis and that excessive SCFA production may paradoxically disrupt the epithelial barrier. Together, these findings suggest the existence of a potential therapeutic window for dietary fiber interventions. Clinical outcomes following fiber intervention are highly context-dependent, influenced by the dosage, the chemical architecture of the fiber, and the host’s baseline inflammation status. Therefore, determining the optimal amount and fiber type for specific endotypes of allergic patients will be important. Further validation in well-controlled clinical studies will be required before such an approach can be implemented.

2. Standardization of Fiber Fractions: Standardized characterization and reporting of fiber properties, e.g., DE/DB for pectin, degree of polymerization distribution for inulin, and molecular size and solubility for β-glucan, are needed to improve the comparability and reproducibility of future studies. Many contradictory findings in studies may partly reflect the use of heterogeneous fiber mixtures that vary by source and processing method. However, achieving such standardization remains a significant challenge, as the physicochemical properties of natural polysaccharides are inherently influenced by their botanical origin and the specific parameters of industrial extraction. Establishing consensus on minimal reporting standards for fiber architecture would represent an important step toward ensuring the reproducibility of clinical and experimental findings.
3. Metabolite Prediction and Modulation: Beyond identifying metabolites like SCFAs, secondary bile acids, and tryptophan metabolites, it will be essential to develop prediction models for immunomodulatory metabolite production based on an individual’s gut microbial profile. Understanding how these metabolites reach distal organs will likely be critical for understanding and treating multi-organ allergic conditions. In this context, deciphering the complex fiber-microbe-metabolite axes will be vital for resolving contradictory outcomes, such as those observed between pectin and inulin. Future research will need to prioritize the precise identification of microbial species and their specific functional genes responsible for producing beneficial secondary bile acids, such as UDCA, isoDCA, or isoalloLCA. Moreover, it will be important to investigate how distinct fiber architectures selectively enrich specific bacterial taxa possessing specialized PULs and bile acid-transforming enzymes, e.g., bile salt hydrolases and 7α-dehydroxylases. Prediction models integrating fiber structure, dosage, and microbial enzymatic capacity may eventually predict whether a specific intervention will shift the metabolite profile toward a tolerogenic state or trigger inflammatory pathways.
4. Limitations: Despite the growing body of evidence supporting the immunomodulatory potential of soluble dietary fibers, several important limitations should also be acknowledged. First, although mechanistic insights have been substantially advanced using murine models, well-designed human clinical trials remain limited. Second, considerable heterogeneity in fiber source, molecular structure, dosage, intervention duration, and study design complicates direct comparison among studies. Third, individual differences in gut microbiome composition are likely to influence the metabolic response to dietary fibers, limiting the generalizability of current findings. Therefore, caution is warranted when translating experimental observations into clinical practice. Finally, because this review synthesizes findings from studies with diverse experimental designs, careful interpretation is required when directly comparing their outcomes or drawing causal conclusions.

In conclusion, although accumulating evidence supports the immunomodulatory potential of dietary fibers, current findings should be interpreted in light of the limited clinical evidence and substantial heterogeneity among studies. Continued validation through standardized experimental protocols and well-designed human trials will be essential to determine the clinical applicability of dietary fiber-based interventions for allergic diseases. While the structural complexity of natural polysaccharides remains a significant challenge, it also provides a vast “molecular library” for the development of targeted dietary interventions. Deciphering the relationship between fiber architecture, microbial metabolism, and immune regulation may facilitate the development of tailored dietary strategies for allergic diseases.

## Key References

- Parrish A, Boudaud M, Grant ET, Willieme S, Neumann M, Wolter M, et al. Akkermansia muciniphila exacerbates food allergy in fibre-deprived mice. Nat Microbiol. Nature Publishing Group; 2023;8:1863–79.
  - ○ This study reveals that dietary fiber depletion in mice triggers an expansion of *Akkermansia muciniphila*, which erodes the intestinal mucus layer for energy. This degradation compromises the gut barrier, allowing food allergens to penetrate more easily and demonstrating experimentally that a specific bacterium promotes the development of food allergies under conditions of dietary fiber deficiency.
- Steigerwald H, Blanco-Pérez F, Macías-Camero A, Albrecht M, Huch M, Bender C, et al. Effects of pectin methyl-esterification on intestinal microbiota and its immunomodulatory properties in naive mice. Carbohydr Polym. 2024;334:122007.
  - ○ This research investigates how the degree of methyl-esterification in pectin influences gut microbiota, their derived metabolites (e.g., SCFAs and bile acids), and immune cells in the lamina propria of naive mice. It highlights that the chemical structure of fibers is a crucial factor in how they modulate microbial communities and the immune system in the gut.
- Gómez F, Pérez-Sánchez N, Macías-Camero A, Núñez R, Contreras N, Cañas JA, et al. Multi-omics analysis of a prebiotic intervention with pectin in lipid transfer proteins (LTPs) allergic patients. Carbohydr Polym. 2025;369:124236.
  - ○ This study presents the first multi-omics comparative analysis of two structurally distinct pectins, low-methoxyl citrus and high-methoxyl apple, within a randomized, double-blind, placebo-controlled (DBPC) intervention for LTP-allergic patients, suggesting pectin’s potential as a structure-specific precision prebiotic for allergy management.
- Arifuzzaman M, Won TH, Yano H, Uddin J, Emanuel ER, Hu E, et al. Dietary fiber is a critical determinant of pathologic ILC2 responses and intestinal inflammation. J Exp Med. 2024;221:e20232148.
  - ○ This study reveals that an inulin-rich diet promotes Tph1-expressing inflammatory ILC2s and eosinophilic inflammation via a microbiota-derived bile acid-IL-33 axis. The findings suggest that high fiber intake may paradoxically exacerbate type 2 inflammation.
- Han K, Xie F, Animasahun O, Nenwani M, Kitamoto S, Kim Y, et al. Inulin-gel-based oral immunotherapy remodels the small intestinal microbiome and suppresses food allergy. Nat Mater. 2024;23:1444–55.
  - ○ This study introduces an inulin-gel-based oral immunotherapy (OIT) that acts as a microbiome-modulating platform to establish sustained unresponsiveness. By normalizing ileal microbiota and enhancing regulatory T cell responses, this engineered gel suppresses pathogenic type 2 immunity, offering a more effective and stable therapeutic approach in OIT.

## Data Availability

No datasets were generated or analysed during the current study.

## Abbreviations

AhR: Aryl hydrocarbon receptor
APCs: Antigen presenting cells
DCs: Dendritic cells
DBPCFC: Double-blind, placebo-controlled food challenge
FA: Food allergy
GPCRs: G-protein coupled receptors
HMP: High methoxyl pectin
ILCs: Innate lymphoid cells
LMP: Low methoxyl pectin
Mφ: Macrophages
MR: Mannose receptor
PRRs: Pattern recognition receptors
PULs: Polysaccharide Utilization Loci
SCFAs: Short chain fatty acids
Tregs: Regulatory T cells

## Declarations

### Experiments on Live Subjects

N/A.
